# Differences in the prevalence of erectile dysfunction between novel subgroups of recent-onset diabetes

**DOI:** 10.1007/s00125-021-05607-z

**Published:** 2021-11-20

**Authors:** Haifa Maalmi, Christian Herder, Gidon J. Bönhof, Klaus Strassburger, Oana-Patricia Zaharia, Wolfgang Rathmann, Volker Burkart, Julia Szendroedi, Michael Roden, Dan Ziegler, GDS Group

**Affiliations:** 1 Institute for Clinical Diabetology, German Diabetes Center (DDZ), Leibniz Center for Diabetes Research at Heinrich Heine University Düsseldorf, Düsseldorf, Germany; 2 German Center for Diabetes Research (DZD), Partner Düsseldorf, München-Neuherberg, Germany; 3 Department of Endocrinology and Diabetology, Medical Faculty and University Hospital Düsseldorf, Heinrich Heine University Düsseldorf, Düsseldorf, Germany; 4 Institute for Biometrics and Epidemiology, German Diabetes Center (DDZ), Leibniz Center for Diabetes Research at Heinrich Heine University Düsseldorf, Düsseldorf, Germany

**Keywords:** Diabetes subgroups, Erectile dysfunction, Inflammation, Insulin resistance, New-onset diabetes

## Abstract

**Aims/hypothesis:**

In men with diabetes, the prevalence of erectile dysfunction increases with advanced age and longer diabetes duration and is substantially higher in men with type 2 diabetes than those with type 1 diabetes. This study aimed to evaluate the prevalence of erectile dysfunction among the five novel subgroups of recent-onset diabetes and determine the strength of associations between diabetes subgroups and erectile dysfunction.

**Methods:**

A total of 351 men with recent-onset diabetes (<1 year) from the German Diabetes Study baseline cohort and 124 men without diabetes were included in this cross-sectional study. Erectile dysfunction was assessed with the International Index of Erectile Function (IIEF) questionnaire. Poisson regression models were used to estimate associations between diabetes subgroups (each subgroup tested against the four other subgroups as reference) and erectile dysfunction (dependent binary variable), adjusting for variables used to define diabetes subgroups, high-sensitivity C-reactive protein and depression.

**Results:**

The prevalence of erectile dysfunction was markedly higher in men with diabetes than in men without diabetes (23% vs 11%, *p* = 0.004). Among men with diabetes, the prevalence of erectile dysfunction was highest in men with severe insulin-resistant diabetes (SIRD) (52%), lowest in men with severe autoimmune diabetes (SAID) (7%), and intermediate in men with severe insulin-deficient diabetes (SIDD), mild obesity-related diabetes (MOD) and mild age-related diabetes (MARD) (31%, 18% and 29%, respectively). Men with SIRD had an adjusted RR of 1.93 (95% CI 1.04, 3.58) for prevalent erectile dysfunction (*p* = 0.038). Similarly, men with SIDD had an adjusted RR of 3.27 (95% CI 1.18, 9.10) (*p* = 0.023). In contrast, men with SAID and those with MARD had unadjusted RRs of 0.26 (95% CI 0.11, 0.58) (*p* = 0.001) and 1.52 (95% CI 1.04, 2.22) (*p* = 0.027), respectively. However, these associations did not remain statistically significant after adjustment.

**Conclusions/interpretation:**

The high RRs for erectile dysfunction in men with recent-onset SIRD and SIDD point to both insulin resistance and insulin deficiency as major contributing factors to this complication, suggesting different mechanisms underlying erectile dysfunction in these subgroups.

**Graphical abstract:**

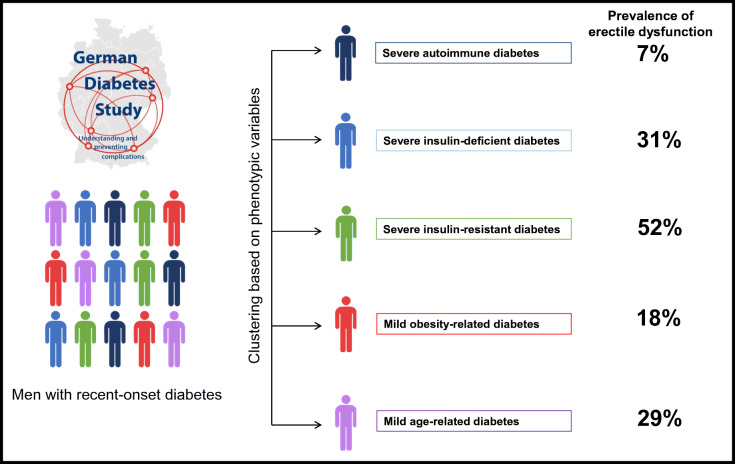

**Supplementary Information:**

The online version contains peer-reviewed but unedited supplementary material available at 10.1007/s00125-021-05607-z.

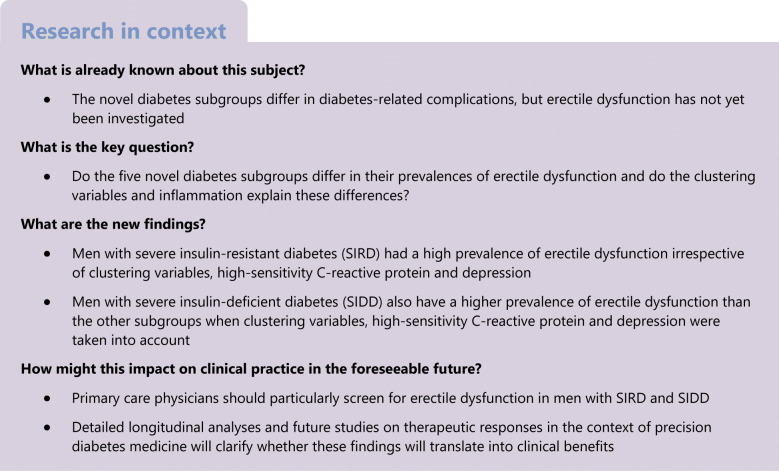



## Introduction

Erectile dysfunction, defined as the persistent inability to obtain or maintain a penile erection during sexual intercourse [1], is a disorder affecting mainly men older than 40 years [2] and is associated with poor relationship quality, low life satisfaction and low self-esteem [3]. In addition, erectile dysfunction is associated with an increased risk of future cardiovascular events, CHD, stroke and all-cause mortality [2, 4, 5].

Men with diabetes are three times more susceptible to develop the condition than men without diabetes [6]. The higher risk of erectile dysfunction in diabetes is associated with higher age, sedentary lifestyle and the presence of the metabolic syndrome (i.e. hyperglycaemia, hypertension and obesity) [7, 8]. Additionally, several studies demonstrated that distal sensorimotor polyneuropathy (DSPN), cardiac autonomic neuropathy (CAN), CVD, non-alcoholic fatty liver disease (NAFLD), depression and hypogonadotropic hypogonadism increase the risk of erectile dysfunction [9–13]. Available epidemiological data report a higher prevalence of erectile dysfunction in men with type 2 diabetes than those with type 1 diabetes. A systematic review and meta-analysis summarising data of 145 studies showed a prevalence of 66% in type 2 diabetes vs 37% in type 1 diabetes [14]. Of note, recent studies using data-driven cluster analyses have refined this established classification of diabetes to include five subgroups that reflect better the heterogeneity of the disease [15, 16]. Therefore, an updated assessment of the prevalence of erectile dysfunction is timely.

Indeed, the novel diabetes subgroups showed a different prevalence of diabetes-related complications. The subgroup designated severe insulin-deficient diabetes (SIDD) has the highest prevalence of retinopathy, DSPN and CAN [15, 16], suggesting, therefore, the possibility of a higher prevalence of erectile dysfunction in SIDD. In addition, the subgroup with severe insulin-resistant diabetes (SIRD) had the highest cardiovascular risk [15] and the highest levels of biomarkers of inflammation, including high-sensitivity C-reactive protein (hs-CRP) [16, 17], which may raise the hypothesis that this subgroup might also exhibit a higher erectile dysfunction prevalence.

Therefore, this study aimed: (1) to assess the prevalence of self-reported erectile dysfunction among novel subgroups of recent-onset diabetes; (2) to assess the strength of the associations between diabetes subgroups and prevalent erectile dysfunction; and (3) to investigate to what extent the clustering variables, hs-CRP and depression explain these associations. Such knowledge could help to understand the underlying mechanisms better and improve effective disease management.

## Methods

### Study design and study population

The German Diabetes Study (GDS) is an ongoing observational prospective study established in 2005 to evaluate the natural course of recently diagnosed diabetes and explore prognostic factors and mechanisms leading to diabetes-related complications [18]. Individuals with known diabetes duration of <1 year and aged 18–69 years at the time of the baseline examination are eligible to participate, while individuals with secondary forms of diabetes, current pregnancy and acute or severe chronic cardiac, hepatic, renal or psychiatric diseases are excluded. Diabetes diagnosis is based on the ADA criteria [19], and diabetes-related autoantibodies are measured in every participant. All participants undergo a comprehensive examination at baseline comprising standardised questionnaires, clinical examinations and detailed laboratory measurements.

The GDS is conducted according to the Declaration of Helsinki, approved by the ethics committee of Heinrich Heine University, Düsseldorf, Germany (ref. 4508) and was registered with ClinicalTrials.gov registration no. NCT01055093. All participants provided written informed consent.

**Fig. 1:**
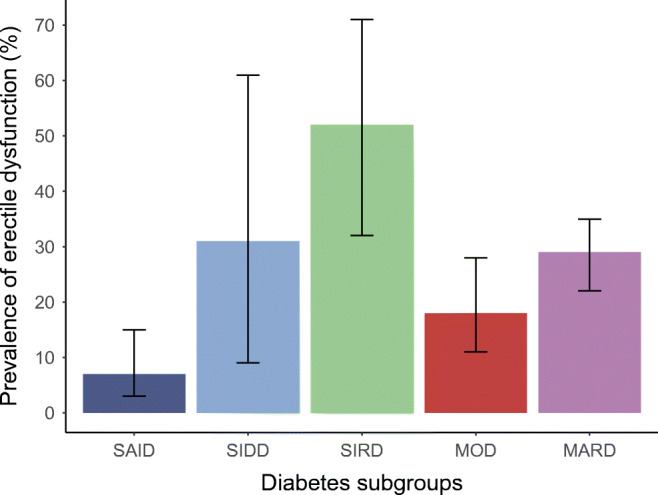
Prevalence of erectile dysfunction across subgroups of recent-onset diabetes from the GDS. Data are given as percentages and corresponding 95% CIs

This cross-sectional analysis focused on 539 consecutive men who were allocated to one of the five diabetes subgroups as part of our previously published data-driven cluster analysis [16]. From this sample, we excluded 188 men (95 men who did not have any sexual attempt during the last 4 weeks and 93 men who did not respond to one or more questions regarding their erectile function), leaving 351 men with diabetes subgroup allocation and complete data on erectile function. The study sample is illustrated in electronic supplementary material (ESM) Fig. 1. This analysis also included 124 men without diabetes from the GDS who have complete data on erectile function. Inclusion criteria for this group were age ≥18 years, normal glucose tolerance and absence of first-degree relatives with diabetes, while exclusion criteria corresponded to those defined for individuals with diabetes.

### Assessment of erectile dysfunction

Erectile dysfunction was defined using questions one to five of the self-reported International Index of Erectile Function (IIEF-5) questionnaire in men who had a least one sexual attempt within the last 4 weeks [20, 21]. The IIEF-5 is a validated tool frequently used to assess erectile dysfunction in epidemiological studies or to assess treatment response in intervention trials. Participants were asked five questions to evaluate their ability to achieve and maintain an erection sufficient for satisfactory sexual intercourse without any treatment. Response options included ‘never’, ‘rarely’, ‘sometimes’, ‘mostly’ and ‘always’ and were ranked from 1 to 5. A total score ranging from 5 to 25 was calculated, with lower IIEF scores indicating poorer erectile function. Men with an IIEF score <22 are considered to have erectile dysfunction.

### Data collection and measurements

The measurement of laboratory variables including HbA_1c_, fasting C-peptide, fasting glucose, HDL-cholesterol, LDL-cholesterol, triacylglycerols and GAD antibodies (GADA) was performed according to standardised laboratory procedures [16, 18]. HOMA2-B and HOMA2-IR were calculated with the HOMA2 calculator based on fasting C-peptide and fasting glucose (https://www.dtu.ox.ac.uk/homacalculator/, accessed 1 Dec 2020). The eGFR was calculated from serum creatinine and cystatin C using the Chronic Kidney Disease Epidemiology (CKD-EPI) equation. Two biomarkers of systemic inflammation (hs-CRP, IL-6) and two biomarkers of vascular inflammation (soluble intercellular adhesion molecule-1 [sICAM-1], soluble E-selectin [sE-selectin]) were measured as described [22, 23]. These four biomarkers were selected because they indicate increased cardiovascular risk and potential endothelial dysfunction [24].

Information on known diabetes duration, anthropometric and lifestyle factors, and on the presence of chronic diseases and medication use (glucose-lowering drugs [insulin/metformin/none/other], non-steroidal anti-inflammatory drugs [yes/no], lipid-lowering drugs [yes/no] and phosphodiesterase-5 inhibitors [yes/no]) was obtained from questionnaires [18]. Prevalent CVD was defined as self-reported myocardial infarction, peripheral arterial occlusive disease, cerebrovascular disease and stroke. Hypertension was defined as systolic BP ≥140 mmHg, diastolic BP ≥90 mmHg or use of antihypertensive medication. Peripheral nerve function was assessed with electrophysiological testing [23]. Neurological examination was performed using the Neuropathy Disability Score (NDS), while neuropathic symptoms were assessed using the Neuropathy Symptom Score (NSS) [25]. DSPN was defined according to the Toronto Consensus criteria [26] based on electrophysiological testing, NDS and NSS as described [23]. Autonomic nerve function was measured with cardiovascular autonomic reflex tests, including heart rate variability indices as described before [27]. CAN was diagnosed if three or more out of seven indices were abnormal [27]. Depressive symptoms were assessed using the Allgemeine Depressionsskala, Langversion (ADS-L), the German version of the internationally validated Center for Epidemiological Studies Depression Scale (CES-D). The ADS-L score ranges from 0 to 60, with a score ≥21 indicating depression [28].

### Statistical analysis

This analysis builds on our previous work [16], which allocated GDS participants to the novel diabetes subgroups using centroids primarily identified in several Scandinavian cohorts [15]. The novel diabetes classification was based on age at diagnosis, BMI, HbA_1c_, HOMA2-B, HOMA2-IR and GADA [15, 16], and led to five subgroups (clusters). The first subgroup is severe autoimmune diabetes (SAID) comprising individuals with type 1 diabetes and determined by the presence of GADA. The other four subgroups, i.e. SIDD, SIRD, mild obesity-related diabetes (MOD) and mild age-related diabetes (MARD), are subtypes reflecting the heterogeneity of type 2 diabetes.

Data are presented as median (25th/75th percentiles) or percentages in descriptive statistics. Differences in characteristics according to erectile dysfunction status, diabetes status and diabetes subgroup allocation were tested with the Mann–Whitney *U* (Wilcoxon) test, the Kruskal–Wallis test and the χ^2^ test. The prevalence of erectile dysfunction is reported using percentages with 95% CIs. Differences in the subgroup distribution and clinical characteristics of included vs excluded participants from the original sample used to perform the cluster analysis were compared with the Mann–Whitney *U* (Wilcoxon) test and the χ^2^ test.

Associations between diabetes subgroups (independent variable) and erectile dysfunction (binary dependent variable) were assessed using Poisson regression models with a robust error variance. Model 1 was unadjusted. Model 2 was adjusted for the clustering variables (age at diagnosis, BMI, HbA_1c_, HOMA2-B, HOMA2-IR and GADA; all co-variables used as continuous variables). Model 3 was additionally adjusted for log_2_-transformed hs-CRP (the only tested biomarker of inflammation that showed a significant difference between men with and without erectile dysfunction). Model 4 was additionally adjusted for depression. First, each diabetes subgroup was tested against the other subgroups as a reference group. Second, the five diabetes subgroups were tested in pairs (ten pairwise associations) accounting for multiple group comparisons by applying the Tukey–Kramer correction in all models. Associations were estimated with RRs of prevalent erectile dysfunction and their corresponding 95% CIs.

Additional analyses included men without diabetes as a reference group. As a sensitivity analysis, we repeated the analysis after excluding participants with prevalent CVD because CVD may represent an intermediate step in the causal pathway between diabetes and erectile dysfunction [9, 10, 29, 30], which might bias our estimates.

All statistical analyses were carried out with SAS version 9.4 (SAS Institute, Cary, NC, USA), and *p* values <0.05 were considered indicators of a statistically significant difference or association.

## Results

### Prevalence of erectile dysfunction in the total study sample

Erectile dysfunction was present in 23% of all men with diabetes (*N* = 351). The characteristics of men with (*n* = 82) and without erectile dysfunction (*n* = 269) are displayed in Table 1. Men with erectile dysfunction were older, had higher BMI values, higher HOMA2-IR and higher triacylglycerols and hs-CRP but lower HDL-cholesterol levels. Also, men with and without erectile dysfunction differed in their use of glucose-lowering drugs and prevalent DSPN but were similar in their glycaemic control, smoking status, eGFR values, hypertension and prevalence of CVD, CAN and depression.

**Table 1:** Clinical characteristics in the total study sample and stratified by erectile dysfunction

			Erectile dysfunction		
Characteristic	*N*	Total sample	Present: *n* = 82 (23%)	Absent: *n* = 269 (77%)	*p*
IIEF score	351	25 (22, 25)	18 (14, 20)	25 (24, 25)	<0.0001
Age (years)	351	49.4 (39.1–57.6)	55.2 (47.5–62.3)	47.5 (36.4–55.5)	<0.0001
BMI (kg/m^2^)	351	28.1 (25.3–32.5)	28.7 (26.6–32.0)	27.5 (25.2–31.4)	0.022
Diabetes subgroups, %	351				<0.0001
SAID		23	7	28	
SIDD		4	5	3	
SIRD		7	17	5	
MOD		25	20	26	
MARD		41	51	38	
Diabetes duration (days)	351	177 (104–262)	185 (110–272)	175 (104–254)	0.587
HbA_1c_ (mmol/mol)	351	44.2 (39.9–51.9)	43.1 (39.9–48.6)	44.2 (39.9–51.9)	0.658
HbA_1c_ (%)	351	6.2 (5.8–6.9)	6.1 (5.8–6.6)	6.2 (5.8–6.9)	0.658
HOMA2-B	351	76.1 (52.0–110.4)	80.0 (60.0–111.9)	74.0 (50.7–110.4)	0.225
HOMA2-IR	351	1.9 (1.2–2.8)	2.2 (1.5–3.7)	1.8 (1.1–2.7)	0.0004
GADA>0.9 units/ml, %	351	23	7	28	0.0001
Current smokers, %	288	27	24	27	0.436
eGFR (ml min^−1^ [1.73 m]−^2^)	324	95 (85–105)	91 (82–103)	96 (85, 105)	0.060
Triacylglycerols (mmol/l)	351	1.36 (0.89–2.02)	1.65 (1.08–2.50)	1.31 (0.84–1.86)	0.0005
HDL-cholesterol (mmol/l)	345	1.16 (0.96–1.39)	1.09 (0.90–1.29)	1.19 (0.98–1.42)	0.011
LDL-cholesterol (mmol/l)	345	3.13 (2.56–3.78)	3.28 (2.64–3.85)	3.10 (2.53–3.75)	0.310
Hypertension, %	350	62	68	60	0.161
CVD, %	344	6	9	5	0.259
DSPN, %	324	16	26	13	0.010
CAN, %	348	4	6	3	0.274
Depression, %	351	10	13	9	0.235
Glucose-lowering drugs, %	346				<0.0001
None		30	34	28	
Metformin		32	52	26	
Insulin		30	10	36	
Other		9	4	10	
Lipid-lowering drugs, %	351	13	13	13	0.854
NSAIDs, %	351	13	13	13	0.854
hs-CRP (nmol/l)	333	19.0 (9.5–28.6)	19.0 (9.5–38.1)	9.5 (9.5–28.6)	0.046
IL-6 (pg/ml)	188	1.5 (1.0–2.3)	1.8 (1.2–2.2)	1.4 (0.9–2.3)	0.084
sICAM-1 (ng/ml)	188	230 (199–272)	223 (198–247)	239 (200–275)	0.148
sE-selectin (ng/ml)	188	41.1 (29.8–52.8)	40.9 (31.7–53.6)	41.2 (29.2–52.6)	0.778

Continuous variables are given as median (25th percentile–75th percentile) and categorical variables are given as percentages (%)

NSAIDs, non-steroidal anti-inflammatory drugs

Men excluded from the analysis (*N* = 188) were older and had a higher BMI, higher HOMA2-IR, lower eGFR and higher hs-CRP than men who were included (*N* = 351), but there were no differences in the other characteristics (ESM Table 1).

As shown in ESM Table 2, men without diabetes (*N* = 124) had a lower prevalence of erectile dysfunction (11%, *p* = 0.004) than men with diabetes.

#### Prevalence of erectile dysfunction across diabetes subgroups

The prevalence of erectile dysfunction among diabetes subgroups is shown in ESM Table 3 and visually illustrated in Fig. 1. The prevalence of erectile dysfunction was highest in SIRD (52%), lowest in SAID (7%), and intermediate in SIDD (31%), MOD (18%) and MARD (29%) (*p* < 0.0001). Diabetes subgroups also showed differences in other baseline characteristics (ESM Table 3). Briefly, men with SAID (*n* = 81, 23%) were younger. Men with SIDD (*n* = 13, 4%) had poorer glycaemic control, lower HOMA2-B and a higher prevalence of CAN. Men with SIRD (*n* = 27, 7%) had pronounced insulin resistance, lower eGFR, higher triacylglycerol levels, more often hypertension and higher hs-CRP. Men with MOD (*n* = 87, 25%) had higher BMI values and men with MARD (*n* = 143, 41%) were older. The subgroups distribution did not differ between included and excluded study participants (ESM Table 1).

#### Associations between diabetes subgroups and erectile dysfunction

The associations between each diabetes subgroup (using the other four subgroups as reference) and erectile dysfunction are shown in Table 2. Men with SIRD had a higher prevalence of erectile dysfunction than men with other diabetes types, and this association remained consistent and statistically significant across the four models (model 4: RR 1.93 [95% CI 1.04, 3.58], *p* = 0.038). Men with SIDD also had a higher prevalence of erectile dysfunction than men with other diabetes types after adjustment for clustering variables in model 2, which remained stable after further adjustment (model 4 (RR 3.27 [95% CI 1.18, 9.10], *p* = 0.023). The most prominent confounders increasing the RR from model 1 to model 2 were age and HOMA2-IR. Men with MARD had a higher (RR 1.52 [95% CI 1.04, 2.22], *p* = 0.027) and men with SAID had a lower (RR 0.26 [95% CI 0.11, 0.58], *p* = 0.001) prevalence of erectile dysfunction in model 1, but these associations did not remain significant in models 2, 3 and 4. No association was observed between MOD and erectile dysfunction.

**Table 2:** Associations between diabetes subgroups and erectile dysfunction

	Model 1		Model 2		Model 3		Model 4	
Diabetes subgroup	RR (95% CI)	*p*	RR (95% CI)	*p*	RR (95% CI)	*p*	RR (95% CI)	*p*
SAID	0.26 (0.11, 0.58)	0.001	0.47 (0.15, 1.41)	0.179	0.44 (0.15, 1.28)	0.132	0.44 (0.15, 1.26)	0.126
SIDD	1.33 (0.57, 3.08)	0.501	2.86 (1.07, 7.59)	0.034	3.12 (1.14, 8.54)	0.026	3.27 (1.18, 9.10)	0.023
SIRD	2.47 (1.62, 3.76)	<0.0001	2.15 (1.14, 4.03)	0.017	2.04 (1.10, 3.79)	0.023	1.93 (1.04, 3.58)	0.038
MOD	0.73 (0.45, 1.20)	0.218	0.76 (0.41, 1.40)	0.386	0.74 (0.40, 1.34)	0.328	0.74 (0.40, 1.33)	0.313
MARD	1.52 (1.04, 2.22)	0.027	0.94 (0.55, 1.61)	0.841	1.02 (0.59, 1.76)	0.917	1.06 (0.61, 1.83)	0.833

Each diabetes subgroup was tested against the four other diabetes subgroups as reference group

Model 1: unadjusted; model 2: adjusted for age, BMI, HbA_1c_, HOMA2-B, HOMA2-IR and GADA; model 3: adjusted for model 2 + log_2_ hs-CRP; model 4: adjusted for model 3 + depression

The results of the associations between each pair of diabetes subgroups and erectile dysfunction are shown in Table 3. In the unadjusted model (model 1), men with SIRD had a higher prevalence of erectile dysfunction than men with MOD. In contrast, men with SAID had a lower prevalence of erectile dysfunction than men with SIRD or MARD. After adjusting for the clustering variables (model 2), men with SIRD had a higher prevalence of erectile dysfunction than men with MOD or MARD, whereas men with SAID had a lower prevalence of erectile dysfunction than men with SIDD or SIRD. However, none of these associations in model 2 remained significant after adjustment for multiple comparisons. After additional adjustment for hs-CRP (model 3) and depression (model 4), RRs for all comparisons remained almost unaltered. In the fully adjusted model 4, men with SIRD still had a higher prevalence of erectile dysfunction than men with MOD (RR 2.21 [95% CI 1.02, 4.79], *p* = 0.043), and men with SAID had a lower prevalence of erectile dysfunction than men with SIDD (RR 0.18 [95% CI 0.04, 0.77], *p* = 0.020) or SIRD (RR 0.23 [95% CI 0.06, 0.83], *p* = 0.025). However, these associations were not statistically significant after adjustment for multiple comparisons.

**Table 3:** Pairwise associations between diabetes subgroups and erectile dysfunction

Subgroup comparison	Model 1			Model 2			Model 3			Model 4		
	RR (95% CI)	*p*	*p*_*Tukey–Kramer*_	RR (95% CI)	*p*	*p*_*Tukey–Kramer*_	RR (95% CI)	*p*	*p*_*Tukey–Kramer*_	RR (95% CI)	*p*	*p*_*Tukey–Kramer*_
SAID vs SIDD	0.24 (0.08, 0.74)	0.013	0.093	0.21 (0.05, 0.88)	0.033	0.205	0.19 (0.05, 0.80)	0.023	0.156	0.18 (0.04, 0.77)	0.020	0.142
SAID vs SIRD	0.14 (0.06, 0.33)	<0.0001	<0.0001	0.20 (0.05, 0.77)	0.019	0.132	0.21 (0.06, 0.77)	0.019	0.129	0.23 (0.06, 0.83)	0.025	0.165
SAID vs MOD	0.40 (0.16, 0.98)	0.045	0.262	0.48 (0.15, 1.55)	0.223	0.740	0.49 (0.16, 1.51)	0.213	0.725	0.51 (0.17, 1.54)	0.233	0.755
SAID vs MARD	0.25 (0.11, 0.57)	0.0009	0.008	0.44 (0.14, 1.40)	0.165	0.636	0.41 (0.13, 1.28)	0.125	0.539	0.41 (0.13, 1.27)	0.122	0.533
SIDD vs SIRD	0.59 (0.24, 1.45)	0.252	0.782	0.95 (0.26, 3.41)	0.935	1.000	1.09 (0.29, 3.95)	0.901	0.999	1.24 (0.33, 4.60)	0.750	0.998
SIDD vs MOD	1.67 (0.66, 4.23)	0.277	0.813	2.26 (0.78, 6.52)	0.131	0.556	2.53 (0.88, 7.30)	0.086	0.422	2.74 (0.93, 8.04)	0.066	0.352
SIDD vs MARD	1.05 (0.44, 2.46)	0.915	1.000	2.06 (0.72, 5.88)	0.178	0.661	2.15 (0.73, 6.34)	0.166	0.638	2.23 (0.74, 6.71)	0.155	0.614
SIRD vs MOD	2.82 (1.59, 4.10)	0.0004	0.004	2.38 (1.01, 5.17)	0.028	0.179	2.33 (1.09, 5.00)	0.029	0.189	2.21 (1.02, 4.79)	0.043	0.257
SIRD vs MARD	1.76 (1.13, 2.75)	0.012	0.088	2.17 (1.05, 4.49)	0.037	0.225	1.98 (0.95, 4.12)	0.068	0.359	1.80 (0.87, 3.72)	0.113	0.508
MOD vs MARD	0.63 (0.37, 1.04)	0.072	0.375	0.91 (0.43, 1.91)	0.804	0.999	0.85 (0.39, 1.80)	0.670	0.993	0.81 (0.38, 1.74)	0.593	0.984

Model 1: unadjusted; model 2: adjusted for age, BMI, HbA_1c_, HOMA2-B, HOMA2-IR and GADA; model 3: adjusted for model 2 + log_2_ hs-CRP; model 4: adjusted for model 3 + depression

The results of the sensitivity analysis are shown in ESM Table 4. After excluding men with prevalent CVD (6%), the associations were consistent with the primary analysis, with the SIRD and SIDD subgroups being associated with a higher prevalence of erectile dysfunction.

The results of the additional analyses are shown in ESM Table 5. Compared with men without diabetes, men with diabetes, particularly men with SIRD and MARD, had significantly higher unadjusted RRs for erectile dysfunction. Although these associations were attenuated after adjustments, the effect estimates for all subgroups combined or analysed separately remained >1 (e.g. RR 1.53 [95% CI 0.85, 2.78] for the comparison of all men with diabetes vs those without diabetes).

## Discussion

This study found a prevalence of erectile dysfunction of 23% in middle-aged men with recent-onset diabetes, which is about double that in men without diabetes. Pathophysiology-based diabetes subgroups showed differences in the prevalence of erectile dysfunction. Men with SIRD had the highest, whereas those with SAID had the lowest erectile dysfunction prevalence. The SIRD and SIDD subgroups showed higher RRs for erectile dysfunction in multivariable models, and these associations were independent of the clustering variables, hs-CRP and depression. In contrast to previous studies on erectile dysfunction in long-standing diabetes, the unique feature of this study is its focus on newly diagnosed disease and the consideration of the heterogeneity of diabetes pathophysiology reflected by the five diabetes subgroups.

The observed erectile dysfunction prevalence of 23% in men with diabetes in GDS was in line with previous reports (20–37%) in men with recent diabetes diagnosis [31–33]. In contrast, most studies conducted in men with longer-term diabetes report a higher prevalence ranging from 35 to 90% [6]. Notably, a meta-analysis of 145 studies in men with longer-term type 1 and type 2 diabetes reported an overall erectile dysfunction prevalence of 52.5% [14]. This variation in the prevalence of erectile dysfunction might be attributed to differences in the study populations (age, ethnicity, diabetes duration) and definition of erectile dysfunction (e.g. single item question or validated multi-item scale) [34, 35]. The lower prevalence of erectile dysfunction in our study may relate to the rather young age of our study population (median age 49.4 years), their short diabetes duration (<1 year), their good health status including good glucometabolic control and low prevalence of CVD and CAN [18], and the use of a multi-item questionnaire to define erectile dysfunction only in sexually active men, unlike several previous studies which used one single item question to assess erectile function regardless of sexual activity.

SIRD, the subgroup characterised by obesity and pronounced insulin resistance, was associated with higher RR for prevalent erectile dysfunction than all the other four subgroups, particularly when compared with MOD, the subgroup characterised by obesity but not by insulin resistance. These associations were robust across models when considering all four subgroups as a reference and in pairwise associations. This finding reinforces that insulin resistance increases the risk of erectile dysfunction [36] and explains the high prevalence of erectile dysfunction in the metabolic syndrome [8, 21], which is often characterised by insulin resistance as a key feature. The molecular mechanism underlying the association between insulin resistance and erectile dysfunction involves endothelial dysfunction in the penile arteries, decreasing their synthesis and release of endothelial nitric oxide. However, it is unclear whether endothelial damage in penile arteries is related to whole-body insulin resistance, which is characterised by decreased responsiveness of the liver, adipose tissue and skeletal muscle to insulin, or is instead related to decreased endothelial insulin sensitivity. Evidence from in vivo studies suggests that endothelium-specific insulin resistance can induce endothelial dysfunction independently of whole-body insulin resistance [37]. In our study, associations between diabetes subgroups and erectile dysfunction were only slightly attenuated after adjustment for HOMA2-IR, indicating that whole-body insulin resistance may only partly explain the observed associations. HOMA2-IR is primarily an index of hepatic insulin resistance but also shows a good correlation with the hyperinsulinaemic–euglycaemic clamp, the ‘gold-standard’ technique for evaluating insulin-stimulated glucose metabolism and whole-body insulin resistance [16, 38]. Of note, SIRD was the subgroup with the lowest HbA_1c_ in our study, which indicates that glycaemic control may not be a major determinant of erectile dysfunction in adults with recent-onset diabetes.

Low-grade systemic inflammation is present in insulin resistance, obesity and type 2 diabetes suggesting that the association between insulin resistance and endothelial dysfunction in erectile dysfunction might be linked to inflammation. Accumulating evidence supports the fact that elevated circulating concentrations of biomarkers of inflammation such as hs-CRP and IL-6, and biomarkers of vascular inflammation such as sICAM-1 and sE-selectin, indicate increased cardiovascular risk and damage to the endothelium [24]. In turn, vascular damage triggers an inflammatory reaction and release of proinflammatory mediators, promoting insulin resistance and endothelial dysfunction [39]. In our study, while serum levels of IL-6, sICAM-1 and sE-selectin did not differ between men with and without erectile dysfunction, we found an increase in hs-CRP levels similar to other studies [40–43].

Because insulin resistance precedes diabetes, the development of erectile dysfunction may have started during the prediabetic stage. Studies assessing erectile dysfunction before and after diabetes diagnosis are needed to clarify these relationships. It is also worth mentioning that our findings in men with recent-onset diabetes cannot be generalised to men with long-standing diabetes who are likely to have longer exposure to chronic hyperglycaemia, dyslipidaemia and hypertension associated with greater risk of diabetes-related complications.

Our study showed higher RRs for prevalent erectile dysfunction in men with SIDD after adjustment for the clustering variables, suggesting that low insulin levels not yet compensated with insulin therapy might be involved in erectile dysfunction in men newly diagnosed with diabetes. However, because diabetes subgroup allocation can change over time as a result of treatment and disease progression, a repeated erectile dysfunction assessment is necessary to confirm this finding. Of note, SIRD and SIDD are different in their clinical presentation. Men with SIRD have a pronounced insulin resistance, but also higher BMI and the highest prevalence of hypertension and impaired kidney function based on eGFR. On the other hand, men with SIDD have the worst glycaemic control and highest prevalence of CAN. The observation that both subgroups appear to have similarly increased prevalence of erectile dysfunction suggests a different mechanism underlying their erectile dysfunction. In SIDD, the more classical concept of glucotoxicity seems to be operative, whereas in SIRD pathomechanisms related to insulin resistance such as lipotoxicity, oxidative stress and low-grade inflammation could contribute to their erectile dysfunction. Indeed, individuals with SIRD also present with a higher degree of dyslipidaemia and markers of inflammation [17]. If confirmed in other studies, these observations would have clinically relevant implications.

Differences in erectile dysfunction between diabetes subgroups reported in our study further support the evidence about differences in complications between novel diabetes subgroups. Ahlqvist et al showed a higher risk for CVD in SIRD, although this was explained by sex and age [15]. Because erectile dysfunction and CVD share endothelial dysfunction as a common feature, our findings consolidate the increased risk of SIRD for vascular diseases. Also, Zaharia et al [16] observed a higher prevalence of NAFLD in SIRD; thus, we hypothesised that a higher prevalence of erectile dysfunction might be found in this subgroup [11]. The mechanism underlying both conditions might involve insulin resistance and, to some extent, low serum testosterone [11, 36, 44–46]. Unfortunately, we did not have information on testosterone levels in our study. Thus, we cannot rule out the potential confounding effect of hypogonadism.

Moreover, findings from the GDS and the Scandinavian cohorts showed a higher prevalence of CKD in SIRD [15, 16]. Given that almost 70% of men with CKD report erectile dysfunction [47], a high prevalence of erectile dysfunction in SIRD was plausible. In addition to the higher prevalence of diabetes complications in SIRD, we previously demonstrated that a higher proinflammatory state also characterises this subgroup [17]. These characteristics make it the subgroup that might benefit from early diagnosis and treatment to prevent complications. In this context, it is interesting that a recent clinical trial showed a reduced incidence of erectile dysfunction in men with type 2 diabetes and high cardiovascular risk treated with the GLP-1 receptor agonist dulaglutide [48]. Given the phenotypic similarities between the study population of this trial and the SIRD subgroup, this drug might be of particular therapeutic benefit for individuals with SIRD.

Comparing the proportions of diabetes subgroups in included vs excluded participants did not indicate any selection bias. However, a certain degree of selection bias was evident when comparing the baseline characteristics. For example, the participants included in the present analysis had a slightly better cardiometabolic risk profile than those who were excluded, which might have attenuated the differences between diabetes subgroups.

The strengths of our study are the inclusion of men with type 1 diabetes and type 2 diabetes and the information on the clustering variables, which allowed us to investigate the prevalence of erectile dysfunction in the novel diabetes subgroups. Furthermore, the study included adults within their first year of diabetes diagnosis so that findings were not confounded by long-term hyperglycaemia and the increasing prevalence of other diabetes-related complications.

Our study also has some limitations that warrant consideration when interpreting the results. Limitations include its sample size (specifically limiting the statistical power of pairwise comparisons between diabetes subgroups and of comparisons with non-diabetic men) and its restriction to men from Germany. Therefore, our findings need validation in studies with larger sample sizes and more diverse populations. In addition, although we used a validated questionnaire to assess erectile dysfunction, our assessment is limited because the IIEF questionnaire only evaluates erectile dysfunction acquired during the last 4 weeks, whereas ‘persistent’ erectile dysfunction can only be confirmed if lasting more than 3–6 months [49], and if complemented by a physical examination. Circulating levels of sex hormones and information on primary or secondary hypogonadism were not available for the study sample, meaning that their analysis in the context of the novel diabetes subgroups was not possible. Another limitation is the potential response bias; as this study involved an outcome related to male sexuality, questions concerning sexual performance may feel too personal for some men and increase the likelihood of not answering.

### Conclusion

Our study shows that novel diabetes subgroups have different prevalences of erectile dysfunction. Men with SIRD, which is characterised by pronounced insulin resistance and an increased inflammatory state, and men with SIDD, which is characterised by insulin deficiency, have the highest RRs for erectile dysfunction. This finding suggests that metabolic risk factors for erectile dysfunction may differ between diabetes subgroups. The high prevalence of erectile dysfunction in SIRD and SIDD corroborates their high risk for diabetes-related complications and calls for comprehensive screening and early treatment in these subgroups. Detailed longitudinal analyses in the GDS and future studies on therapeutic responses in the context of precision diabetes medicine will clarify whether these findings will translate into clinical benefits.

## Supplementary Information

ESM (PDF 156 kb)

## Data Availability

The data are subject to national data protection laws. Therefore, data cannot be made freely available in a public repository. However, data can be requested through an individual project agreement with the Steering Committee of the GDS (speaker: Michael Roden, michael.roden@ddz.de).

## Abbreviations

ADS-L: Allgemeine Depressionsskala, Langversion
CAN: Cardiac autonomic neuropathy
CES-D: Center for Epidemiological Studies Depression Scale
CKD: Chronic kidney disease
DSPN: Distal sensorimotor polyneuropathy
GADA: GAD antibodies
GDS: German Diabetes Study
hs-CRP: High-sensitivity C-reactive protein
IIEF: International Index of Erectile Function
MARD: Mild age-related diabetes
MOD: Mild obesity-related diabetes
NAFLD: Non-alcoholic fatty liver disease
NDS: Neuropathy Disability Score
NSS: Neuropathy Symptom Score
SAID: Severe autoimmune diabetes
sE-selectin: Soluble E-selectin
sICAM-1: Soluble intercellular adhesion molecule-1
SIDD: Severe insulin-deficient diabetes
SIRD: Severe insulin-resistant diabetes
