# Supplementary material for: Differences in the prevalence of erectile dysfunction between novel subgroups of recent-onset diabetes
Source: Diabetologia. 2021 Nov 20;65(3):552–62. doi: 10.1007/s00125-021-05607-z (PMC8803719; doi:10.1007/s00125-021-05607-z)
Supplement: Supplementary file 1 — (PDF 156 kb) [file 125_2021_5607_MOESM1_ESM.pdf]

ESM Table 1. Characteristics of included vs excluded participants

**Characteristic**	**Included** N=351	**Excluded** N=188	***p***
Diabetes subgroup, %			0.319
SAID	23%	18%	
SIDD	4%	5%	
SIRD	7%	11%	
MOD	25%	28%	
MARD	41%	38%	
Age (years)	49.4 (39.1; 57.6)	52.1 (43.9; 60.7)	**0.007**
BMI (kg/m^2)	28.1 (25.3; 32.5)	30.0 (25.9; 34.8)	**0.005**
Diabetes duration (days)	177 (104; 262)	153 (99; 259)	0.372
HbA1c (mmol/mol)	44.2 (39.9; 51.9)	45.3 (41.0; 51.3)	0.075
HbA1c (%)	6.2 (5.8; 6.9)	6.3 (5.9; 6.9)	0.075
HOMA2-B	76.1 (52.0; 110.4)	79.7 (53.3; 117.3)	0.417
HOMA2-IR	1.9 (1.2; 2.8)	2.2 (1.3; 3.1)	**0.010**
GADA >0.9 units/ml, %	23%	18%	0.229
Current smokers, %	27%	23%	0.207
eGFR (ml/min per 1.73m^2)	95 (85; 105)	89 (77; 100)	**0.0002**
Triacylglycerols (mmol/L)	1.36 (0.89; 2.02)	1.33 (0.98; 2.01)	0.677
HDL cholesterol (mmol/L)	1.16 (0.96; 1.39)	1.16 (0.98; 1.32)	0.456
LDL cholesterol (mmol/L)	3.13 (2.56; 3.78)	3.05 (2.46; 3.75)	0.367
Hypertension, %	62%	68%	0.143
CVD, %	6%	7%	0.850
Glucose-lowering drugs,%			0.314
None	30%	32%	
Metformin	32%	36%	
Insulin	30%	22%	
Other	9%	10%	
Lipid-lowering drugs, %	13%	16%	0.243
NSAIDs, %	13%	15%	0.402
hs-CRP (nmol/l)	9.5 (9.5; 28.6)	19.0 (9.5; 38.1)	**0.008**
Interleukin-6 (pg/ml)	1.45 (1.0; 2.3)	1.6 (1.0; 2.3)	0.296
sICAM1 (ng/ml)	230 (199; 272)	239 (199; 267)	0.604
sE-selectin (ng/ml)	41.1 (29.8; 52.9)	39.2 (29.0; 53.1)	0.575

Abbreviations: *BMI, body mass index; CVD, cardiovascular diseases; eGFR, estimated glomerular filtration rate; GADA, glutamic acid decarboxylase antibodies; HbA1c, haemoglobin A1c; HDL, high-density lipoprotein; hs-CRP, high-sensitivity C-reactive protein; LDL, low-density lipoprotein; MARD, mild age-related diabetes; MOD, mild obesity-related diabetes; NSAIDs, nonsteroidal anti-inflammatory drugs; SAID, severe autoimmune diabetes; sE-selectin, soluble E-selectin; sICAM-1, soluble intercellular adhesion molecule-1; SIDD, severe insulin-deficient diabetes, SIRD, severe insulin-resistant diabetes.*

Continuous variables are given as mean median (25th percentile, 75th percentile), categorical variables are given as percentages (%).

ESM Table 2. Clinical characteristics of men with and without diabetes

	Men with diabetes		Men without diabetes		*p*
**Characteristic**	**N**	**Median (Q1, Q3)**	**N**	**Median (Q1, Q3)**	
Erectile dysfunction, %	351	23%	124	11%	0.004
IIEF score	351	25 (22; 25)	124	25 (24; 25)	0.056
Age (years)	351	49.4 (39.1; 57.6)	124	45.4 (30.3; 55.1)	0.004
BMI (kg/m^2)	351	28.1 (25.3; 32.5)	124	26.8 (24.8; 29.5)	0.008
Diabetes subgroups, %	351		-	-	-
SAID		23%	-	-	
SIDD		4%	-	-	
SIRD		7%	-	-	
MOD		25%	-	-	
MARD		41%	-	-	
Diabetes duration (days)	351	177 (104; 262)	-	-	-
HbA1c (mmol/mol)	351	44.2 (39.9; 51.9)	117	33.3 (31.1; 35.5)	<0.0001
HbA1c (%)	351	6.2 (5.8; 6.9)	117	5.2 (5.0; 5.4)	<0.0001
HOMA2-B	351	76.1 (52.0; 110.4)	115	121.3 (96.8; 144.9)	<0.0001
HOMA2-IR	351	1.9 (1.2; 2.8)	115	1.2 (0.8; 1.6)	<0.0001
GADA>0.9 units/ml, %	351	23%	124	0%	<0.0001
Current smokers, %	288	27%	124	24%	0.009
eGFR (ml/min per 1.73m^2)	324	95 (85; 105)	110	94 (86; 105)	0.914
Triacylglycerols (mmol/l)	351	1.36 (0.89; 2.02)	118	2.74 (1.89; 4.29)	0.085
HDL cholesterol (mmol/l)	345	1.16 (0.96; 1.39)	118	1.29 (1.08; 1.52)	0.0001
LDL cholesterol (mmol/l)	345	3.13 (2.56; 3.78)	118	3.23 (2.61; 3,90)	0.327
Hypertension, %	350	62%	123	46%	0.003
CVD, %	344	6%	124	1%	0.017
DSPN, %	324	16%	75	0%	<0.0001
CAN, %	348	4%	81	0%	0.082
Depression, %	351	10%	124	4%	0.040
Glucose-lowering drugs, %	346		115		<0.0001
None		30%		100%	
Metformin		32%		0%	
Insulin		30%		0%	
Other		9%		0%	
Lipid-lowering drugs, %	351	13%	124	2%	0.0003
NSAIDs, %	351	13%	124	17%	0.255
hs-CRP (nmol/l)	333	19.0 (9.5; 28.6)	118	9.5 (9.5; 19.0)	<0.0001
Interleukin-6 (pg/ml)	188	1.5 (1.0; 2.3)	-	-	-
sICAM1 (ng/ml)	188	230 (199; 272)	-	-	-
sE-selectin (ng/ml)	188	41.1 (29.8; 52.8)	-	-	-

Continuous variables are given as median (25th percentile, 75th percentile) and categorical variables are given as percentages (%).

NSAIDs, non-steroidal anti-inflammatory drugs.

ESM Table 3. Clinical characteristics according to diabetes subgroups

**Characteristic**	**SAID**	**SIDD**	**SIRD**	**MOD**	**MARD**	***p***
N (% of study sample)	81 (23%)	13 (4%)	27 (7%)	87 (25%)	143 (41%)	
Erectile dysfunction, n (%)	6 (7%)	4 (31%)	14 (52%)	16 (18%)	42 (29%)	<0.0001
IIEF score	25 (25, 25)	25 (17, 25)	21 (18, 25)	25 (23, 25)	24 (20, 25)	<0.0001
Age (years)	34.8 (27.9; 45.0)	37.0 (29.5; 45.0)	52.9 (49.1; 62.9)	44.3 (36.3; 49.2)	57.3 (51.7; 61.9)	<0.0001
BMI (kg/m^2)	25.1 (22.5; 27.3)	24.7 (22.7; 27.6)	33.1 (30.6; 36.2)	33.1 (30.4; 38.0)	27.3 (25.7; 29.3)	<0.0001
Diabetes duration (days)	157 (109; 243)	83 (53; 114)	190 (123; 272)	175 (110; 268)	189 (109; 261)	0.0332
HbA1c (mmol/mol)	44.2 (41.0; 51.9)	67.2 (58.4; 78.1)	42.1 (39.9; 45.3)	45.3 (38.8; 53.0)	43.1 (39.9; 48.6)	<0.0001
HbA1c (%)	6.2 (5.9; 6.9)	8.3 (7.5; 9.3)	6.0 (5.8; 6.3)	6.3 (5.7; 7.0)	6.1 (5.8; 6.6)	<0.0001
HOMA2-B	47.8 (37.0; 67.8)	30.4 (24.8; 42.0)	172.0 (149.2 (223.4)	91.7 (73.9; 125.2)	78.1 (58.5; 106.7)	<0.0001
HOMA2-IR	0.9 (0.7; 1.3)	1.2 (1.0; 1.7)	4.4 (3.7; 5.2)	2.7 (1.9; 3.4)	1.9 (1.4; 2.5)	<0.0001
GADA >0.9 units/ml, %	100%	0%	0%	0%	0%	<0.0001
Current smokers, %	29%	40%	20%	45%	20%	0.0005
eGFR (ml/min per 1.73m^2)	102 (94; 108)	112 (105; 114)	84 (66; 102)	100 (84; 108)	91 (83; 100)	<0.0001
Triacylglycerols (mmol/l)	0.92 (0.66; 1.34)	2.19 (0.89; 2.32)	2.20 (1.51; 3.71)	1.60 (1.14; 2.24)	1.35 (0.93; 1.95)	<0.0001
HDL cholesterol (mmol/l)	1.37 (1.13; 1.70)	1.34 (1.09; 1.56)	0.93 (0.77; 1.00)	1.03 (0.88; 1.19)	1.19 (1.00; 1.42)	<0.0001
LDL cholesterol (mmol/l)	2.94 (2.50; 3.46)	3.18 (3.05; 3.49)	3.07 (2.56; 3.70)	3.20 (2.66; 4.08)	3.25 (2.59; 3.88)	0.169
Hypertension, %	36%	54%	81%	66%	71%	<0.0001
CVD, %	1%	8%	4%	6%	10%	0.162
DSPN, %	9%	38%	16%	16%	18%	0.106
CAN, %	0%	15%	0%	6%	5%	0.029
Depression, %	11%	15%	22%	8%	8%	0.182
Glucose-lowering drugs, %						<0.0001
None	11%	8%	29%	32%	41%	
Metformin	7%	25%	67%	37%	37%	
Insulin	79%	67%	4%	17%	11%	
Other	3%	0%	0%	14%	11%	
Lipid-lowering drugs, %	2%	15%	22%	7%	20%	0.0005
NSAIDs, %	5%	15%	22%	11%	16%	0.084
hs-CRP (nmol/l)	9.5 (9.5; 19.0)	9.5 (9.5; 28.6)	40.9 (20.0; 53.3)	25.7 (11.4; 47.6)	14.3 (6.7; 33.3)	<0.0001

Interleukin-6 (pg/ml)	1.0 (0.7; 1.8)	1.2 (0.7; 1.5)	2.1 (1.6; 2.9)	1.7 (1.2; 2.4)	1.4 (1.0; 2.3)	0.0002
sICAM1 (ng/ml)	241 (209; 273)	203 (142; 269)	247 (223; 271)	247 (208; 286)	217 (191; 260)	0.088
sE-selectin (ng/ml)	40.9 (29.1; 49.5)	28.2 (24.3; 41.1)	46.2 (42.8; 53.6)	47.1 (37.6; 60.3)	38.4 (28.3; 46.8)	0.002

Continuous variables are given as median (25th percentile, 75th percentile) and categorical variables are given as percentages (%).

ESM Table 4. Associations between diabetes subgroups and erectile dysfunction in men without history of cardiovascular diseases

	Model 1		Model 2		Model 3		Model 4	
**Diabetes subgroup**	RR (95% CI)	*p*	RR (95% CI)	*p*	RR (95% CI)	*p*	RR (95% CI)	*p*
SAID	0.27 (0.12, 0.60)	0.001	0.49 (0.16, 1.50)	0.215	0.47 (0.16, 1.40)	0.177	0.47 (0.16, 1.36)	0.164
SIDD	1.11 (0.40, 3.02)	0.837	2.92 (0.94, 9.02)	0.063	3.38 (1.06, 10.70)	0.038	3.50 (1.09, 11.28)	0.035
SIRD	2.71 (1.77, 4.13)	<0.0001	2.49 (1.29, 4.79)	0.006	2.45 (1.28, 4.68)	0.006	2.27 (1.18, 4.36)	0.014
MOD	0.69 (0.41, 1.18)	0.179	0.72 (0.38, 1.37)	0.321	0.71 (0.38, 1.34)	0.298	0.71 (0.38, 1.33)	0.290
MARD	1.58 (1.06, 2.36)	0.024	0.92 (0.51, 1.65)	0.795	0.96 (0.53, 1.74)	0.895	1.00 (0.55, 1.85)	0.982

Each diabetes subgroup was tested against the four other subgroups as reference group

Model 1: unadjusted.
Model 2: adjusted for age, BMI, HbA1c, HOMA2-B and HOMA2-IR and GADA.
Model 3: adjusted for model 2 + $\log_2$ hs-CRP.
Model 4: adjusted for model 3 + depression.

ESM Table 5. Associations of diabetes and diabetes subgroups with erectile dysfunction

	**Model 1**		**Model 2**		**Model 3**		**Model 4**	
**Comparison**	RR (95% CI)	*p*	RR (95% CI)	*p*	RR (95% CI)	*p*	RR (95% CI)	*p*
SAID *vs* controls	0.60 (0.24, 1.51)	0.286	1.58 (0.42, 5.84)	0.489	1.54 (0.59, 4.03)	0.380	1.51 (0.55, 4.17)	0.424
SIDD *vs* controls	2.52 (0.97, 6.54)	0.056	1.89 (0.10, 33.44)	0.663	2.39 (0.13, 42.62)	0.554	2.17 (0.09, 48.70)	0.625
SIRD *vs* controls	4.26 (2.31, 7.84)	<0.0001	1.96 (0.74, 5.17)	0.173	2.23 (0.86, 5.78)	0.098	1.83 (0.67, 5.00)	0.238
MOD *vs* controls	1.51 (0.78, 2.92)	0.221	1.81 (0.55, 5.97)	0.327	1.53 (0.46, 5.05)	0.486	1.38 (0.41, 4.62)	0.597
MARD *vs* controls	2.41 (1.38, 4.19)	0.002	1.49 (0.69, 3.21)	0.308	1.66 (0.77, 3.56)	0.196	1.63 (0.75, 3.54)	0.211
All subgroups vs controls	1.92 (1.13, 3.24)	0.015	1.48 (0.82, 2.67)	0.192	1.59 (0.88, 2.87)	0.122	1.53 (0.85, 2.78)	0.156

Each diabetes subgroup was tested against men without diabetes (controls) as reference group

Model 1: unadjusted.
Model 2: adjusted for age, BMI, HbA1c, HOMA2-B and HOMA2-IR and GADA.
Model 3: adjusted for model 2 + $\log_2$ hs-CRP.
Model 4: adjusted for model 3 + depression.

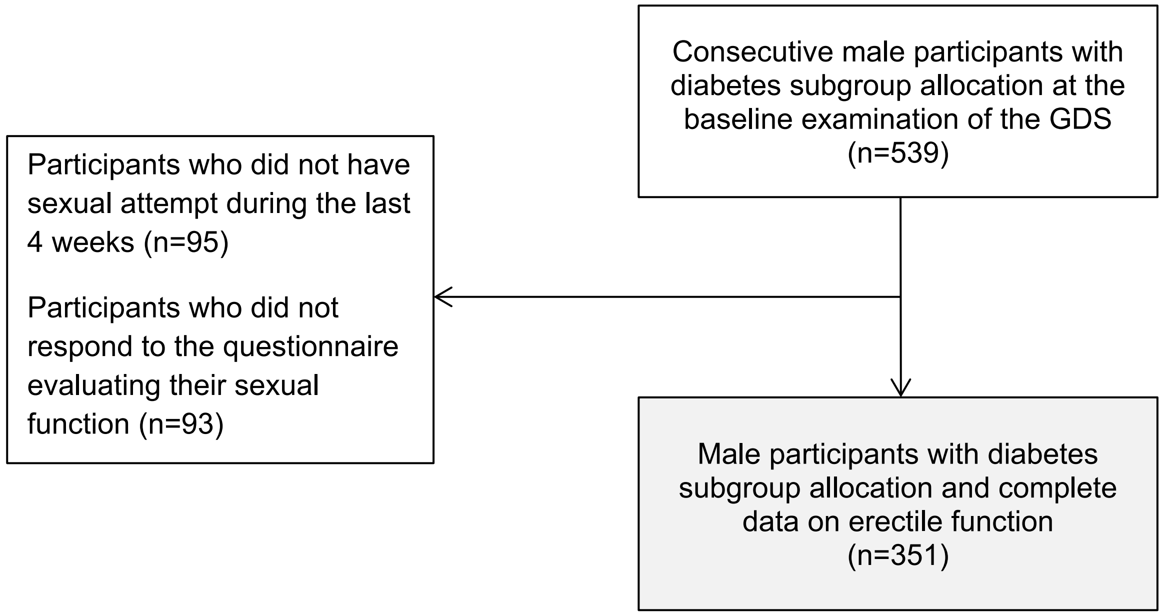

ESM Fig. 1. Flow chart of the study sample
